# The perceptions of university students on technological and ethical risks of using robots in long-term care homes

**DOI:** 10.3389/frobt.2023.1268386

**Published:** 2023-12-21

**Authors:** Erika Young, Lillian Hung, Joey Wong, Karen Lok Yi Wong, Amanda Yee, Jim Mann, Krisztina Vasarhelyi

**Affiliations:** ^1^ UBC IDEA Lab, School of Nursing, University of British Columbia, Vancouver, BC, Canada; ^2^ McGill University, Faculty of Medicine and Health Sciences, Montreal, QC, Canada; ^3^ Vancouver Coastal Health Research Institute, Vancouver, BC, Canada

**Keywords:** telepresence robot, person-centered care, older adult, social connection, gerontechnology, long-term care, students, ethical risk

## Abstract

**Introduction:** The COVID-19 pandemic has disproportionately impacted long-term care (LTC) residents and exacerbated residents’ risks of social isolation and loneliness. The unmet emotional needs of residents in LTC have driven researchers and decision-makers to consider novel technologies to improve care and quality of life for residents. Ageist stereotypes have contributed to the underuse of technologies by the older population. Telepresence robots have been found to be easy to use and do not require older adults to learn how to operate the robot but are remotely controlled by family members. The study aimed to understand the perspectives of multidisciplinary university students, including healthcare students, on using telepresence robots in LTC homes. The study would contribute to the future planning, implementation, and design of robotics in LTC.

**Methods:** Between December 2021 and March 2022, our team conducted interviews with 15 multidisciplinary students. We employed a qualitative descriptive (QD) approach with semi-structured interview methods. Our study aimed to understand the perspectives of university students (under the age of 40) on using telepresence robots in LTC homes. Participants were invited to spend 15 min remotely driving a telepresence robot prior to the interview. A diverse team of young researchers and older adults (patient and family partners) conducted reflexive thematic analysis.

**Results:** Six themes were identified: Robots as supplementary interaction; privacy, confidentiality, and physical harm; increased mental well-being and opportunities for interactions; intergenerational perspectives add values; staffing capacity; environmental and cultural factors influence acceptance.

**Conclusion:** We identified a diverse range of perspectives regarding risk and privacy among participants regarding the implementation of telepresence robots in long-term care. Participants shared the importance of the voice of the resident and their own for creating more equitable decision-making and advocating for including this type of technology within LTC. Our study would contribute to the future planning, implementation, and design of robotics in LTC.

## Introduction

Social isolation is a risk factor for depression and anxiety (Domènech-Abella et al., 2019). There are multiple risk factors for social isolation unique to older adults living in long-term care (LTC), (e.g., lack of independence, language barrier, and being disconnected from family and friends (Boamah et al., 2021). Lacking regular human contact and social interaction can result in cognitive decline for older adults (Sharkey and Sharkey, 2012; Lara et al., 2019). The COVID-19 pandemic has disproportionately impacted LTC residents and exacerbated residents’ risks of social isolation and loneliness. Across the Organisation for Economic Co-operation and Development (OECD) countries, 40% of total COVID-19 deaths were in LTC since the pandemic started in 2020 to April 2021 (Organisation for Economic Co-operation and Development). Policies and measures to limit or eliminate in-person visits during the pandemic socially isolated residents from their family members, friends, and volunteers (Chu et al., 2021). The unmet emotional needs of residents in LTC have driven researchers and decision-makers to consider novel technologies to improve care and quality of life for residents.

Gerontechnology, as defined by Bronswijk and colleagues, is “an interdisciplinary field that links existing and developing technologies to the aspirations and needs of aging and aged adults” (Bronswijk et al.). Robotic companion dogs and cats that provide meaningful activities and positive experiences for residents (Fogelson et al., 2021), and an interactive digital designed for older adults to allow social connection via video calls, pictures, and text messages are examples of gerontechnology (Badawy et al., 2022).

Technology offers significant benefits for older adults, and also poses potential risks or unintended consequences for LTC residents (Boissy et al., 2007; Nylander et al., 2012; Cesta et al., 2013; Niemelä et al., 2017; Reis et al., 2018; O Brolchain, 2019; Vandemeulebroucke et al., 2020; Isabet et al., 2021; Tan et al., 2021; Hung et al., 2022; Lolich et al., 2022; Mariano et al., 2022; Shin et al., 2022). Issues frequently discussed in both conceptual and empirical literature include the reduction in human contact and concerns about privacy and safety (Boissy et al., 2007; Nylander et al., 2012; Cesta et al., 2013; Niemelä et al., 2017; Reis et al., 2018; O Brolchain, 2019; Isabet et al., 2021; Tan et al., 2021; Hung et al., 2022; Shin et al., 2022). O’Brolchain discussed that the dominance of technology would replace a proportion of meaningful human relationships that are based on physical presence and in-person communication (O Brolchain, 2019). Healthcare workers expressed worries about family members monitoring residents through robots (Niemelä et al., 2017). Furthermore, researchers explored inequalities and ageism. Mariano and colleagues suggested that the ageist stereotypes contributed to the underuse of technology in the older population (Mariano et al., 2022). This point is supported by healthcare staff’s concerns towards the unfamiliarity of older adults with technology (Boissy et al., 2007; Isabet et al., 2021) as they observed the interactions of older adults with devices like smartphones and computers in nursing homes (Lolich et al., 2022).

The existing body of literature primarily addresses the perception of gerontechnology focuses on reporting the perspectives of staff, family, and older adults (Boissy et al., 2007; Nylander et al., 2012; Cesta et al., 2013; Niemelä et al., 2017; Reis et al., 2018; Vandemeulebroucke et al., 2020; Verloo et al., 2020; Isabet et al., 2021; Hung et al., 2022; Lolich et al., 2022; Mariano et al., 2022; Shin et al., 2022). However, a limited number of articles delve into the perspectives of emerging healthcare professionals and students with diverse specializations, such as future physicians, nurses (van Kemenade et al., 2018; Lukasik et al., 2020), occupational therapy students (Kristoffersson et al., 2011; Tobis et al., 2017), and those majoring in industrial design (Huang and Liu, 2019), specifically concerning the application of robotics in healthcare and for older adults.

In a study by Van Kemenade and colleagues, healthcare students demonstrated greater acceptance of companion robots compared to assisting and monitoring robots (van Kemenade et al., 2018). Nursing and medical students criticized that companion robots should never replace human interactions (Lukasik et al., 2020), while occupational therapy students emphasized the role of robots as therapy aides, clarifying that they should complement rather than replace healthcare professionals or family members (Tobis et al., 2017). Occupational therapy students also commented on the safety aspect of the robots’ mobile function (Kristoffersson et al., 2011). Lukasik and colleagues reported healthcare professional students’ concerns about the preparedness and difficulties of older adults to handle and use robots (Lukasik et al., 2020), while students from industrial design stated that older adults’ traditional beliefs would affect their understanding of robots (Huang and Liu, 2019).

There is value in considering the perspective of students in different disciplines. For example, nursing students focused more on the positive robotic functions of enhancing social connections and reducing loneliness, while medical students were concerned about privacy issues (Lukasik et al., 2020). We argue that the gap in knowledge on student perspectives on gerontechnology across a range of disciplines should be addressed as current students will be future leaders who will influence policies in healthcare and other corresponding fields, as well as being future caregivers to older adults residing in LTC. Given the rapid evolution of AI and robotics in healthcare, these students are likely to come across ethical considerations in different fields impacting gerontechnology such as engineering, communications, business, and design. Therefore, it is beneficial to foster a comprehensive understanding of gerontechnology among students, understanding their perspectives to navigate and contribute ethically to the evolving landscape.

**FIGURE 1:**
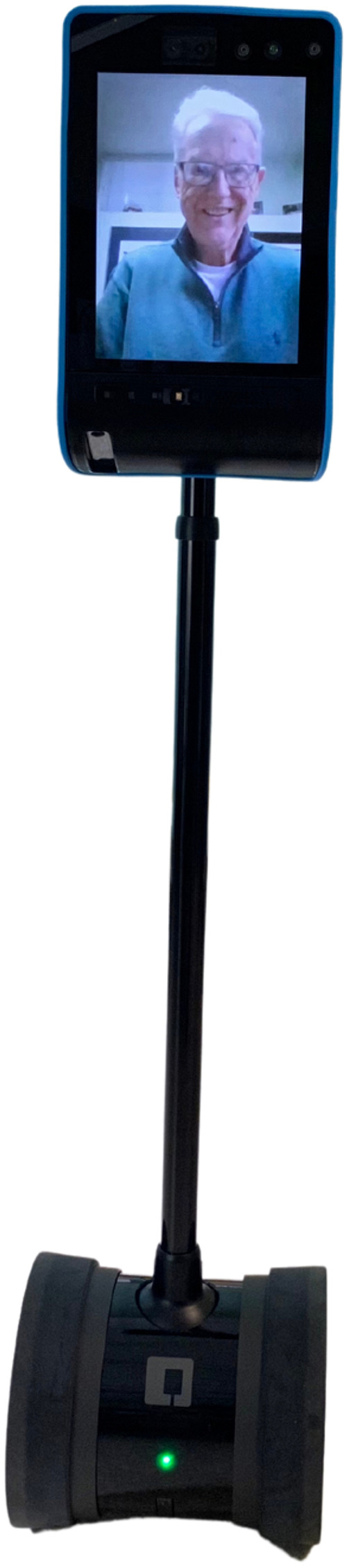
A telepresence robot.

The telepresence robot (Figure 1) is a technology being explored in LTC recently to address social isolation by facilitating virtual connections between residents and family members. These robots are video-conferencing devices on wheels that allow real-time communication and movement on command. Family members can remotely drive and control the robots to visit the residents in LTC via a wireless connection to the internet (Hung et al., 2022). Literature showed that telepresence robots were easy to use and did not require older adults to learn how to operate the robot (Moyle et al., 2014; Koceski and Koceska, 2016; Korblet, 2019). However, both family members and care workers were concerned about privacy and residents’ control over the calls via the robots (Niemelä et al., 2017). An occupational therapy student stated that telepresence services of robots would be a supplement in providing care, other than a replacement of healthcare staff or family members (Tobis et al., 2017). However, like other technologies used in LTC, there is limited research on students’ perceptions of adopting telepresence robots in LTC.

Our study aimed to understand the perspectives of multidisciplinary university students (under the age of 40) on using telepresence robots in LTC homes. Our study would contribute to the future planning, implementation, and design of robotics in LTC.

## Methods

Between December 2021 and March 2022, we conducted interviews with 15 people. We employed a qualitative descriptive (QD) approach with semi-structured interview methods (Sandelowski, 2000). QD design is well suited for this inquiry as the study’s aim was to exploring young university students’ perceptions of robot use in an older population. QD helps fill the research gap of previous literature by gaining personal insights into “what concerns and matters” in the context of LTC homes.

### Recruitment

We used a purposive sampling method to recruit five male and ten female, undergraduate and graduate students undertaking studies in health, life sciences or technology-related fields and with representing diverse racial backgrounds (Table 1). The inclusion criteria are university students and 35 years of age or younger. We asked participants to help us to invite other student informants from any recognized Canadian university. After 15 people were interviewed, we gained sufficient information to answer the study questions. Detailed participants’ characteristics are shown in Table 1.

**TABLE 1:** Participant characteristics.

Participant	Grad/Undergrad	Field of study	Identified gender	Age range	Cultural background
1	Undergrad	Science, Behavioural Neuroscience	F	19–25	Chinese
2	Undergrad	General Science	M	19–25	Filippino and Chinese
3	Undergrad	Applied Animal Biology	F	19–25	United States
4	Undergrad	Political Science and International Relations	F	19–25	Mexican
5	Undergrad	Nursing	F	19–25	Chinese
6	Graduate	Engineering, Clean Energy	M	30–35	South Asian
7	Undergrad	Nursing	F	19–25	Chinese, First Nation, European
8	Undergrad	Psychology	M	19–25	Chinese Malaysian
9	Undergrad	Computer Science	F	19–25	Chinese
10	Graduate	Political Science	F	30–35	Caucasian
11	Undergrad	Computer Science and Math	M	19–25	East African
12	Undergrad	Neuroscience and Pharmacology	F	19–25	Caucasian
13	Undergrad	Social Work	M	30–35	Caucasian
14	Undergrad	Engineering	F	19–25	Asian
15	Undergrad	Engineering	F	19–25	Southeast Asian

### Data generation

Participants were given a link to a 2-min video of how the robot works prior to the interview. Also, they were invited to spend 15 min remotely driving a telepresence robot (Double Robotics) prior to the interview to gain a sense of the user experience. The interviews were conducted by Zoom meeting and lasted 30–40 min in a space of the participant’s choosing. We used live transcription and audio recording over Zoom. The interview questions are listed in Table 2.

**TABLE 2:** Interview questions.

Questions	Prompts
Can you think of any concerns or benefits involving this type of technology (telepresence robots) in long-term care?	Do you have concerns around technological risks?
	If you placed yourself in the position of a resident, would there be any concerns? How about a staff member? What would be the benefits to staff from using this technology?
Imagine your family member is living in LTC. What would you think would be their experience? Why would you think this?	Would you have any concerns for your family member?
	Would you support the use of this type of technology? Would your peers?
	What would you think would be the attitude or acceptance of the robot with older adults you know?
	Have you much experience teaching your grandparents or older adults on technology before COVID? How about during COVID?
What are some circumstances and factors that may affect accessibility of use?	How would we determine the eligibility for access just when thinking that residents in long-term care have varying levels of physical and cognitive impairments?
Do you think older adults should be supported to use more technologies for social connection in LTC?	What are some ways that individuals can be supported?

### Ethics consideration

Ethical approval for this study was obtained from the University of British Columbia Ethics Review Board. An informed consent form was signed by each participant and collected electronically. We offered each participant a CAD $20 gift card from a local grocery store in appreciation of their contributions.

### Data analysis

The interview transcripts were analyzed using reflexive thematic analysis to generate themes that described the stories of participants’ perspectives and opinions (33). The analysis process involved: dataset familiarization, data coding, initial theme generation, theme development and review, theme refining, defining, and naming, and writing up. Three authors performed the first three steps. We conducted team discussion to complete the rest of the steps. The team analysis was conducted by two Zoom meetings. Before the meetings, the lead author (EY) ensured all team members had access and reviewed the interview data and preliminary findings to facilitate a more productive discussion. The older person with lived experience (JM) helped interpret the interview results and challenged taken-for-granted assumptions. Students from disciplines of Nursing, Social Work, Medicine, and Pharmacy, brought diverse perspectives. This multidisciplinary approach led to a more comprehensive understanding of the themes. Researchers (LH, KV) guided the process by supporting the interpretations made by the team, ensuring that the findings were scientifically sound. To ensure scientific rigor in this qualitative research, we applied a reflexive team approach throughout the study to critically examine our analysis process and individual assumptions. We recognized that the researchers’ positioning inevitably shapes the way of interpretation of data. The diverse perspectives of the research team members (patient partners, academic researchers, and older and younger people) were encouraged and valued. The team discussion helped reflect on our thinking and forced us to describe how and why interpretations were formed. To support the credibility of the findings, themes were discussed repeatedly with the whole team, a process that generated revisions and refinements for quality. Reflexivity embedded in our research meetings deepened and enriched the complex analysis, which supported not only scientific rigor but also transparency. We included patient and family partners in the analysis, which helped to challenge our assumptions and taken-for-granted knowledge by looking for various interpretations of the data and encouraging each other to “dig a little deeper”, building on our in-depth practice knowledge and background to identify aspects of the themes that might otherwise have gone un-noticed.

## Results

**FIGURE 2:**
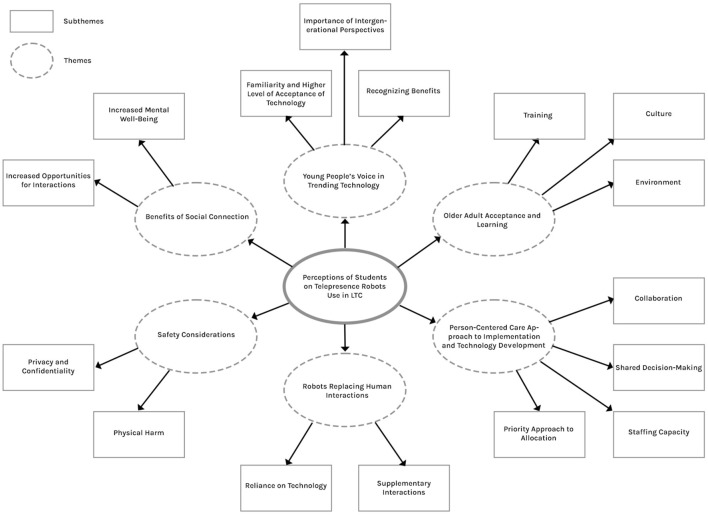
Thematic map.

Participants noted older adults’ willingness to accept technology in LTC for social connection, emphasizing the need for technical training. Sociocultural factors, such as familial bonding importance, influenced acceptance, requiring staff and family support. A person-centered approach, considering autonomy and collaboration, was deemed essential. Challenges included technology allocation, language accessibility, and adapting to diverse capabilities, requiring staff capacity and design adaptability. Mixed opinions arose on whether robots could replace human interactions, emphasizing a person-centered mindset. Safety concerns included physical risks and privacy issues, but participants believed these concerns could be addressed. Although concerns exist, participants expressed the benefits of using robots in LTC for maintaining social connections and improving mental wellbeing. Participants expressed overall support, citing potential benefits and the need for diverse perspectives. A thematic map was developed to provide an overview of themes and subthemes (Figure 2).

### Older adults’ acceptance and learning

Based on interactions with their grandparents and older adults, participants expressed how older adults are willing to use and accept technology in LTC as a medium for socially connecting older adults with their families.

Many participants noted that technical training would be necessary to help older adults develop a positive attitude and instill confidence when using the robot. Participants expressed how older adults’ living and social environment in the past and present can shape how they use and accept robots. In particular, having opportunities where older adults are trained in using robots or any technology can foster a digitally stimulating environment, which can promote older adults’ acceptance and technological literacy.

“[Older adults] would just need a tutorial [on] how to use the device so whether it be like an iPad or laptop [to help] navigate the telepresence robot through their device” (Participant 2, Male, General Science undergraduate student)

Furthermore, participants discussed how certain sociocultural factors can influence LTC end-users’ utilization of robotics. Notably, participants believed that cultural differences regarding the importance of familial bonding can be a critical determinant in older adult’s and their family’s enthusiasm towards using the robot to maintain social connections. Also, participants pointed out that having staff members who are trained in using the technology and family members supporting the older adults in using the robot:

“I would say that family bonding is a bit higher in [Asian countries], rather than North America. So, at also less technologically savvy people there in that culture” (Participant 6, Male, Engineer in Clean Energy graduate student)

“Something simple [like using technology] can seem really foreign to [older adults] because they're not used to it, but if there is […] support from staff members who are there to help [older adults] navigate, how to use [the robot], and [their] family members are also like really enthusiastic about using it I think it could like have a really good outcome.” (Participant 1, Female, Behavioral Neuroscience undergraduate student)

### Person-centered care

Participants emphasized that the development and use of robots for older adults should be approached using a person-centered mindset. This includes collaborating with older adults and other stakeholders of varying ages to inform technology development and implementation:

“It's always important to see what everyone [at] every age group thinks about [the robot], because everyone kind of grew up in a different technological time. so there may be different opinion[s] on [robotic-related] challenges that [older adults] might come up with [that] I do not think of, or I might think of pros and cons that someone else might not come up with.” (Participant 5, Female, Nursing undergraduate student)

Participants also discussed how the implementation of technology should support older adults’ autonomy and be part of the decision-making process on whether to use the robot. Many participants raised the potential implications of having to use robots for social connections, as it could negatively impact older adults’ psychosocial wellbeing or be too overwhelming. So, participants expressed how older adults, if possible, should be able to decide on when to start, end, or reschedule a video call:

“If [the older adult is] able to express that they do not want to talk or do not want to go on the call […] I do not think necessarily [to] schedule the [robot] but I think just making sure that they do have the option to refuse to join the call or just like request that it's moved to a different time” (Participant 12, Female, Neuroscience and Pharmacology undergraduate student)

Furthermore, challenges in allocating technologies in LTC were noted. Many participants suggested that the use of technology should be prioritized for older adults whose families are unable to visit them in the care home, “emergency” situations, or the level of assistance required. Nevertheless, participants still maintained that despite a priority approach to allocation in LTC, older adults should have the final decision in terms of requesting or declining calls.

Additionally, a key factor in terms of the implementation and design of the robot was the ability to reach older adults with a diverse range of functioning. The participants believed that regardless of older adults’ physical, cognitive, or communication abilities, all residents should be provided with the opportunity to try the robot. Moreover, participants raised the concern that language needs to be considered so more residents can use the robot. For instance, when introducing the robot and how to use it, the instructions should be provided in the resident’s preferred language. Likewise, they noted that technology should be adaptable to the various capabilities of older adults. As highlighted by one of the participants:

“I know sometimes residents have a really hard time hearing so I do not know like volume-wise like how loud [can the robot’s speaker] go […] [or if there is an] option […] like subtitles or something, just so that [the older adult] can like see [what is being said and] it might make [the communication] a bit easier” (Participant 12, Female, Neuroscience and Pharmacology undergraduate student)

In addition, to support the use of the robot in LTC across a range of residents, participants expressed how the technical aspect of deploying a robot in LTC will require staffing capacity. The use of the robot could create more staff burden, as it may require upkeep, experience malfunction, or older adults and their families require assistance when using the robot:

“I do not really know how the process is for them to set up or facilitate this [robot], but I think […] [there is] always a concern […] [of] understaffing.” (Participant 7, Female, Nursing undergraduate student)

“The maintenance of [the robot could require staff] to go around the building, and [the staff will] have to find where [the] robots were.” (Participant 11, Male, Computer Science and Mathematics undergraduate student)

### Robot replacing human interactions?

There were discussions during the interviews about whether robots would replace human interactions. Participants had diverse opinions on this. Some participants raised the concern that robots may replace human interactions.

“We human beings are very social creatures, so my concern would be if we're replacing human interaction and human presence and solely relying on technology.” (Participant 13, Male, Social Work student)

Some participants think that robots should not replace human interactions and should be used as a supplement in addition to human interactions only, when in-person visitation by family and friends is not possible.

“Let's use (the robot) more as a supplement. It's in addition to everything else (human interactions).” (Participant 10, Male, Political Science undergraduate student)

“.When people (family and friends) are busy or there are restrictions when visitors cannot physically be there. Then, at least having sort of video or voice call will be more beneficial than having in my opinion.” (Participant 5, female, Nursing undergraduate student)

However, some participants do not think robots replacing human interactions is a concern. They do not think that robots will replace human interactions.

“I feel like people (family and friends) who want to be physically there would try to be there.” (Participant 7, Female, Nursing undergraduate student)

Some participants had experience volunteering or working in long-term care and think that there are opportunities for residents to interact with staff, so robots replacing human interaction is not a concern.

“I do not think I would be concerned about it, like, replacing face-to-face interaction because I just know like from my personal experience that there is lots of interaction with staff.” (Participant 12, Female, Neuroscience and Pharmacology undergraduate student)

### Safety considerations

There were also discussions on safety considerations. Again, participants had different views on this. Participants generally raised two types of safety concerns about using robots in long-term care. The first type is physical concerns that the robots may run over people.

“If you're in a care facility and somebody's walking around, you probably do not want to run over their foot or something.” (Participant 11, Male, Computer Science and Math undergraduate student)

“If the patient has some mobility or visual disability. that makes them (robots) like accidentally bumped into someone.” (Participant 14, Female, Engineering undergraduate student)

Another type of concern is privacy/confidentiality, for example, private conversations being heard or stored.

“A concern would be confidentiality if a family member accidentally drives it (the robot) into another room or out of the room or something like that.” (Participant 12, Female, Neuroscience and Pharmacology undergraduate student)

“I think some platforms take your information while you're on the video. They store the information.” (Participant 9, Female, Computer Science undergraduate student)

However, most participants were not too worried about these concerns. They think that these concerns can be addressed.

“(For physical concern,) there are sensors (in the robot), so I do not think that’s really a concern.” (Participant 7, Female, Nursing undergraduate student)

“For privacy (concern,) I guess some enclosed environment or something like that (would help).” (Participant 7, Female, Nursing undergraduate student)

### Benefits of social connections

We found general support for the use of robots in long-term care because the benefits of using the robots for social connections, especially during COVID-19, were recognized among our study participants.

“Given like the circumstances of the pandemic, this (the robot) seems to be very useful and almost necessary … In times where families want to see their relatives in long-term care, this provides an easy viable option.” (Participant 4, Female, Political Science and International Relations undergraduate student)

“I think it (using robots in long-term care) would be really nice. They (family) might live further away or have immune system issues and it's hard to go see them (residents) in person frequently. But feeling lonely for a while is not good.” (Participant 3, Female, Applied Animal Biology undergraduate student)

Some participants added that social connections help the mental wellbeing of residents.

“It's really beneficial for mental wellbeing, especially during the pandemic I'm not sure if a lot of people are allowed to visit. I think this is like a really good way to get seniors in the long-term homes, to be able to talk to their family and friends, even during the pandemic.” (Participant 9, Female, Computer Science undergraduate student)

“For the benefits, I do think it’s great for like maintaining familiar social interactions with families, which I think is really important because just having that familiar social interaction can definitely improve like mental health.” (Participant 12, Female, Neuroscience and Pharmacology undergraduate student)

### Young people’s voices in trending technology

Many participants said they think that their peers will support the idea of having a robot in long-term care. They think that they will be able to see and appreciate the benefits of having robots.

“Most of my peers, especially in engineering, are welcome to use technology. Our job is like using technology to make our lives easier and improve our quality of life. This (robot) definitely moves in the direction of improving our convenience and improving our quality of life. So, I think my peers will support this.” (Participant 15, Female, Engineering student)

“I think, a lot of them (peers) would see like the benefits of having this available.” (Participant 7, Female, Nursing undergraduate student)

Some think the discussion on the use of robots in long-term care should include perspectives of different age groups.

“I think it's always important to see what every age group thinks about it (use of robots in long-term care), because everyone grew up in different times, so we all have varying opinions. So, there may be different opinions on the challenges (on the use of robots in long-term care) that they might come up with.” (Participant 5, Female, Nursing undergraduate student)

Some participants think that young people should be included in this discussion because they think that they are more accepting of new technology and thus can tell older adults the benefits of using the robots.

“Like our (younger) generation is definitely more acceptable to new technologies and using them. Some older adults might have like some concerns about adopting new technology. This generation can tell them the benefits of using those technologies.” (Participants 15, Female, Engineering undergraduate student)

“I think it’s important to include the perspective of a new generation because the world is changing, I feel every new generation has a different perspective on things, especially technology, so they have like different ideas that could help like positively impact the healthcare system.” (Participant 9, Female, Computer Science undergraduate student)

“I think generally speaking, we (young people) are very familiar with like technology as a whole. So, I think it is useful input.” (Participant 12, Female, Neuroscience and Pharmacology undergraduate student)

## Discussion

This study explores the perspective of young, diverse university students to better understand perceptions around technological and ethical risks of using robots, specifically telepresence robots, within LTC. Previous literature focused on students in health disciplines and industrial design while also focusing mainly on quantitative data (Tobis et al., 2017; van Kemenade et al., 2018; Lukasik et al., 2020) with few collecting qualitative data (Kristoffersson et al., 2011; Huang and Liu, 2019). This study sought to understand the diversity of perspectives by using a qualitative descriptive approach utilizing semi-structured interviews. This study found that the students had an overall positive perception of older adults’ acceptance and learning of the telepresence robot. These students believed that person-centered care for those living in LTC should be enacted, including during the decision-making and processes for implementation. There was an agreement that telepresence robots could be beneficial to support social connections for residents in LTC. With minimal safety concerns and mixed concerns about the replacement of human interactions, students believed the inclusion of intergenerational voices would be valuable in understanding key benefits in implementation.

### Self-continuity in younger generations: Ethical perceptions and decision-making

When reflecting on experiences, participants talked about their parents living in the community and did not relate this situational context to themselves or their direct family members (unless they had otherwise stated they had family within LTC). These results show a disconnect as they did not relate to future aging populations, nor did they project the futures of themselves or their parents. In other studies, young adults have been found to have an optimistic future vision of self as they believed ‘old’ to apply to their late 20s (Ryff, 1991). When given a specific age of reference (i.e., age 85) they had more realistic aging expectations of a decline in cognitive and physical health (Kornadt and Rothermund, 2014). The participants’ answers demonstrate an implicit ageist mindset as they did not envision themselves or their parents within the context of a LTC setting. Therefore, the participants lack self-continuity. This can be explained by the age difference where participants have comparably fewer years to reflect than those aged 60+; age is positively correlated with self-continuity and the ability to place oneself in a future context (Hershfield, 2011). ‘They do not know what they do not know’ is apparent as the interview question did not give the concept of age, which if provided, has shown to be an easier linkage to the perception of a future self (Kornadt and Rothermund, 2014). The participants therefore may have had issues conceptualizing themselves with physical and cognitive decline as they were unable to relate their current independence and abilities in a congregate setting such as LTC.

With their understanding of technology, there were few concerns found regarding privacy and safety. In a study done by Hundley and Shyles, it was found that young adult participants were aware of these risks of breach of privacy or identity theft, however, spoke about it casually and held an acceptance that this was a possibility when using technology (Hundley and Shyles, 2010). Similarly, our study showed that safety around the use of the robot was very minimal, where student participants had trust in the technology to protect the privacy and confidentiality of the users, which contradicts conceptual and empirical literature examining common ethical concerns in gerontechnology (Tan et al., 2021).

### Person-centered decision-making and the power divide

Most participants stated that they had helped an older adult (parent, grandparent) with the use of technology during the COVID-19 pandemic. It is possible that after seeing and experiencing the isolation firsthand, students had a higher degree of acceptance and consideration for all individuals with cognitive and physical impairments to have a chance to try the robot and evaluate it on a case-by-case basis. This is in contrast to the findings of Lukasik and colleagues where some students showed reluctance that older adults may not be ready to use this type of technology (Lukasik et al., 2020). It should also be noted that on the individual level, participants felt residents should have the choice to utilize this technology, however, when asked about the inclusion of younger people’s voices in power, that the voices of older adults were not included on a mezzo and macro level. The divide between people of power and the inhabitants of these LTC homes themselves was evident as it was only mentioned that staff, policymakers, leaders, and organizations should have a say in decision-making and policy development. On the micro level, healthcare professionals have the knowledge, and a comprehensive understanding of the system, power and control is unbalanced as they must make snap decisions often leading them to make decisions on behalf of residents and patients (McCormack, 2001). Knowledge is often seen as power, which was demonstrated to be a common understanding by the student participants in this study. These beliefs create an imbalance of power in decision-making over the care needs of the residents living in LTC.

Participants noted that within this decision-making process, the older adults living in LTC receiving the call should have the choice to decline the call when families may decide to connect virtually. Within LTC, it is often that family members will drop in throughout the day unannounced. As the robot only has the option to End Call, participants suggested further improvements that can allow for stronger autonomy, such as having the option to call back at a later time. In this context, autonomy, and agency within a LTC setting provides the power to the resident themselves rather than disempowering them (Holstein and Gubrium, 2000). This is important because LTC has many boundaries within its setting such as those resulting from staff shortages and limited resources which result in often overlooking individual needs and leaving power to staff and that of the policies and procedures within the organization, limiting the autonomy and choice of those living in LTC.

### Perceptions of equity and resource allocation

Previous studies have concluded that there has been a negative shift in acceptance of companion robots by healthcare students, in terms of replacing their jobs (van Kemenade et al., 2018). As our results are taken from differing demographics that looked at healthcare students and students in other disciplines, their thoughts and perceptions showed no concern over this fact. These perceptions may be due to envisioning persons in their field (i.e., Engineers, and computer scientists) where they may not feel as threatened if their jobs were to be behind the development of such technologies.

Participants did have the perception that it would reduce staff workload except in instances of technical failures or repositioning the robot when needed. This observation aligns with existing literature which similarly highlighted formal and informal caregiver perceptions regarding the burden placed on the operator in control of the robot (Cesta et al., 2013; Isabet et al., 2021). There were few concerns over staff and technological resources. Participants did not mention the current barriers that exist within this setting and the technical inequities that currently exist, such as paid private Wi-Fi, cable TV, and telephone providers that currently need further payment from the resident or family. The accessibility of the telepresence robot was not as high a concern compared to creating an environment for the comfort of use. These varying thoughts present a different understanding as it is the participants’ understanding of the staffing and resources available within a LTC environment. With participants being unaware of these barriers within LTC, their voices to advocate for these changes can be lost.

### Why the voices of young people matter

As health systems engage in quality improvement initiatives, technology innovations play an important role in moving this sector forward. It is useful to understand the perspectives of a young student demographic as they can influence upcoming policy and disbursement of funding from local governments, while also being future caregivers to older adults. The current student body will include future developers of robotics, healthcare workers, and local politicians. As noted in this study, the younger generation has grown up in the age of technology and is more likely to have a greater understanding of the benefits which allows health systems to be better positioned to harness innovation and new technologies. The World Health Organization has implemented a youth-focused initiative in healthcare while acknowledging the invaluable contributions of the younger generation, affirming their role as both the changemakers of tomorrow and the driving force of today. Current leaders in the field recognize that they should be listening to their insights and ideas, understanding that it is through their perspectives that meaningful progress can be achieved (WHO).

Individual LTC organizations are under legislation and are regulated by local governments and policymakers. As research in technology and healthcare continues to inform best practices, solutions cannot be left strictly to the individual LTC organization to make decisions around the implementation of such technologies. This study emphasizes the need to consider the perspectives of younger generations in the discourse surrounding technology integration as technology implementation within healthcare is a lengthy process and decision-making will be influenced by diverse stakeholders. These changes also include much hesitancy from staff who are used to things a certain way; the preference is to have user-friendly technologies to allow the nurses to focus more on patients rather than the complexities of the technology themselves (Mahoney, 2011).

### Strengths and limitations

This research was strengthened by a transdisciplinary approach, including academic and frontline team members, and a team member living with dementia. An intergenerational team also helped to strengthen the research by providing varying insights. This collaboration helped to enrich data analysis. A reflexive methodology allowed the team to share knowledge and experience, allowing subjectivity to add richness to the thematic analysis. As most studies have previously used a quantitative approach, this study fills a research gap to better explore the context and understanding of the participants’ thought processes.

This study also provided a trial of a telepresence robot. This experience provided participants with a greater understanding of how the telepresence robot worked from the perspective of the family, where it allowed them to control the robot from their computer, tablet, or phone. This study was completed during the COVID-19 pandemic, limiting data collection to be done over Zoom. Virtual interviews resulted in some Wi-Fi technical issues and the inability to see the telepresence robot in person. They were shown a mirror of how they were controlling the robot from their end, however, the experience may have been different if they had seen the technology in person, as it would have given them the element of design and expanded the concept of usage, as attitudes improved with more knowledge of the robot (Johansson-Pajala et al., 2019). Purposive sampling enabled diversity in the participants in terms of interdisciplinary studies, ethnicity, gender, and age allowing for diverse perspectives.

Finally, participants in this study were not directly asked whether they had previous experience or knowledge of the LTC system. Participants may have had altered perceptions of what LTC is skewing the overall perceptions of risk, safety, and equity.

As the field of robotics advances, future research can benefit from a comparative analysis of generational differences among stakeholders. Having a deeper understanding may help to facilitate the development of comprehensive user interfaces that seamlessly integrate ethical considerations, user-friendliness, adaptability, and acceptance.

## Conclusion

Our findings reveal a diverse range of perspectives among the younger generation regarding the implementation of telepresence robots in long-term care. Key considerations include risk and privacy, while also showing considerations towards literacy and the use of technology. The students also raised the importance of voices, their own and those of the residents, to provide a platform for shared decision-making and advocacy towards the decisions of including technology such as social robots to provide care to residents living in LTC. Future research should examine how sex and gender, socioeconomic status ethnic and racial backgrounds, and disciplinary knowledge may shape students’ attitudes and perceptions of robotic use in aged care.

## Data Availability

The original contributions presented in the study are included in the article/Supplementary material, further inquiries can be directed to the corresponding author.
